# The effects of baicalin on piglets challenged with *Glaesserella parasuis*

**DOI:** 10.1186/s13567-020-00826-5

**Published:** 2020-08-14

**Authors:** Shulin Fu, Ronghua Yin, Sanling Zuo, Jun Liu, Yunfei Zhang, Ling Guo, Yinsheng Qiu, Chun Ye, Yu Liu, Zhongyuan Wu, Yongqing Hou, Chien-An Andy Hu

**Affiliations:** 1 Hubei Key Laboratory of Animal Nutrition and Feed Science, Wuhan Polytechnic University, Wuhan, 430023 People’s Republic of China; 2 Hubei Collaborative Innovation Center for Animal Nutrition and Feed Safety, Wuhan, 430023 People’s Republic of China; 3 Biochemistry and Molecular Biology, University of New Mexico School of Medicine, Albuquerque, NM 87131 USA

**Keywords:** baicalin, *Glaesserella parasuis*, piglets, inflammatory response, protection

## Abstract

*Glaesserella parasuis* (*G. parasuis*) causes porcine vascular inflammation and damage. Baicalin is reported to have antioxidant and anti-inflammatory functions. However, whether baicalin protects piglets against *G. parasuis* challenge and the potential protective mechanism have not been investigated. Therefore, in this study, we comprehensively examined the protective efficacy of baicalin in piglets challenged with *G. parasuis* and the possible protective mechanism. Our results show that baicalin attenuated the release of the inflammation-related cytokines interleukin (IL) 1β, IL6, IL8, IL10, and tumour necrosis factor α (TNF-α) and reduced high mobility group box 1 (HMGB1) production and cell apoptosis in piglets infected with *G. parasuis*. Baicalin also inhibited the activation of the mitogen-activated protein kinase (MAPK) signalling pathway and protected piglets against *G. parasuis* challenge. Taken together, our data suggest that baicalin could protect piglets from *G. parasuis* by reducing HMGB1 release, attenuating cell apoptosis, and inhibiting MAPK signalling activation, thereby alleviating the inflammatory response induced by the bacteria. Our results suggest that baicalin has utility as a novel therapeutic drug to control *G. parasuis* infection.

## Introduction

*Glaesserella parasuis* (*G. parasuis*), which belongs to the family Pasteurellaceae, is responsible for Glässer’s disease in pigs. The typical characteristics of Glässer’s disease are fibrinous polyserositis, arthritis, meningitis, and frequent symptoms of pneumonia [1]. To date, 15 serovars have been identified with the heat-stable antigen extraction method [2], but a significant percentage of isolates has not been classified by this method [3]. Serovar 5 is thought to be highly virulent, and epidemiological studies have shown that serovars 4, 5, and 13 are the current epidemic strains in China [3]. With changes in the breeding patterns in pigs, the economic losses caused by *G. parasuis* have become more severe. Because there are numerous serovars of *G. parasuis* that do not induce protective cross-immunity, the prevention and control of *G. parasuis* infection have become urgent problems [4].

The pathogenic mechanism of *G. parasuis* is currently unclear, so it is difficult to control infection. However, several virulence-related factors have recently been reported to be involved in its pathogenicity [5, 6]. *G. parasuis* induces autophagy in the porcine alveolar macrophage (PAM) cell line 3D4/21 via the mitogen-activated protein kinase (MAPK) signalling pathway [7]. Additionally, deletion of the *rfaD* and *rfaF* genes involved in lipooligosaccharide (LOS) biosynthesis of *G. parasuis* decreases the secretion of proinflammatory cytokines in PAMs through regulation of the nuclear factor κB (NF-κB) and MAPK signalling pathways during the infection process [8]. Previous research has shown that the AI-2/luxS quorum sensing system affects the growth and virulence of *G. parasuis* [9]. The bacterium also disrupts adherens junctions and initiates the epithelial–mesenchymal transition, leading to fibrinous polyserositis, which depends on the regulation of the canonical WNT/β-catenin signalling pathway [10]. QseC-mediated osmotic stress resistance and biofilm formation regulate the density of *G. parasuis* [11]. These virulence-related factors not only are important pathogenic factors but also elicit the host immune response [12] and are therefore considered as important drug targets for the prevention of Glässer’s disease.

With the extensive use of antibiotics on pig farms, the phenomenon of bacterial resistance has become more serious. Therefore, screening for environmentally friendly efficacious drugs for which resistance has not been developed has become an urgent focus of disease control. Baicalin, extracted from *Scutellariae Radix*, has been shown to have potential therapeutic functions. Previous research has demonstrated that baicalin relieved the inflammation in L-02 and THLE2 liver cells induced by lipopolysaccharide (LPS) by upregulating taurine upregulated 1 (TUG1) [13]. Baicalin attenuated the intestinal disruption caused by challenge with *Salmonella pullorum* and modulated the gut microbiota in laying hens [14]. It also alleviated the inflammatory immune responses of chicken type II pneumocytes stimulated with avian pathogenic *Escherichia coli* (APEC) and may inhibit APEC biofilm formation and the expression of APEC virulence genes [15]. Baicalin also improved the health of mice and prevented their infection with *Helicobacter pylori* in a model of inflammation by interfering with the growth and virulence of *H. pylori* [16]. Baicalin protected mice against *S. Typhimurium* challenge by modulating both the bacterium’s virulence and the host’s immune response [17]. In our previous work, we found that baicalin could suppress the NF-κB and NLRP3 inflammasome signalling pathways induced by *G. parasuis* in porcine aortic vascular endothelial cells (PAVECs) [18] and piglet mononuclear phagocytes (PMNPs) [19]. Baicalin reduced apoptosis triggered by *G. parasuis* via RAGE, MAPK, and AP-1 in PAVECs [20]. Baicalin also inhibited PKC-MAPK signalling pathway activation [21] and attenuated high-mobility group box 1 (HMGB1) secretion [22] in PMNPs stimulated by *G. parasuis*. In addition, baicalin could modulate long non-coding RNA and mRNA expression in PAVECs infected by *G. parasuis* [23]. However, whether baicalin can protect piglets against *G. parasuis* challenge has not been investigated.

In this study, we investigated the effects of baicalin in piglets challenged with *G. parasuis*. Our results suggest that baicalin has utility as a novel therapeutic drug to control *G. parasuis* infection in pigs.

## Materials and methods

### Bacterial strain and growth conditions

*Glaesserella parasuis* strain SH0165 serovar 5 was isolated from the lung of a commercially produced pig with the typical characteristics of Glässer’s disease, including arthritis, fibrinous polyserositis, haemorrhagic pneumonia, and meningitis [24]. The SH0165 isolate was cultured at 37 °C for 12 h in tryptic soy broth (Difco Laboratories, USA) or grown for 24 h in tryptic soy agar (Difco Laboratories) supplemented with 10 μg/mL nicotinamide adenine dinucleotide (Sigma, USA) and 10% foetal bovine serum (Gibco, Australia).

### Drugs

Baicalin was provided by the National Institutes for Food and Drug Control (Beijing, B110715-201318). Before use, baicalin was dissolved and diluted with RPMI-1640 medium (Gibco, USA).

Ethyl pyruvate (EP) and flunixin meglumine (FM) were purchased from Shanghai Macklin Biochemical Co., Ltd. and Guangdong WenS Dahuanong Biotechnology Co., Ltd., respectively.

### Animals and experimental design

Fifty-six 30-day-old naturally farrowed early-weaned piglets (Duroc × Landrace × Large White) weighing 8–10 kg were purchased from Wuhan Wannianqing Animal Husbandry Co., Ltd. (Wuhan, China) for the in vivo experiments. The piglets used in this study were weaned on day 23. The piglets were negative for antibodies directed against *G. parasuis* when tested with INgezim Haemophilus 11.HPS.K.1 (INgezim, Spain).

The 56 piglets were randomly divided into seven groups of eight piglets each: the negative control group, infection group, EP group, FM group, treatment group 1, treatment group 2, and treatment group 3. Before *G. parasuis* challenge, the piglets in the EP group were injected intraperitoneally with EP at 40 mg/kg body weight (BW); the piglets in the FM group were injected intramuscularly with FM at 2 mg/kg BW; and the piglets in treatment group 1, treatment group 2, and treatment group 3 were injected intramuscularly with baicalin at 25, 50, and 100 mg/kg BW, respectively. After 30 min, the infection group, EP group, FM group, treatment group 1, treatment group 2, and treatment group 3 were challenged intraperitoneally with 1 × 10^9^ CFU of the *G. parasuis* strain in 2 mL of normal saline. The piglets from the negative control group were injected intraperitoneally with an equivalent volume of saline. The piglets in all the groups were monitored for 7 days after challenge, and their morbidity and mortality were recorded.

### Analysis of cytokine secretion and HMGB1 with enzyme-linked immunosorbent assay (ELISA) and real-time quantitative PCR (qPCR)

At 48 h following *G. parasuis* challenge, blood samples were collected for cytokine analysis. The presence of interleukin (IL) 1β, IL6, IL8, IL10, tumour necrosis factor α (TNF-α), and HMGB1 in the sera was detected with ELISA kits (for IL1β, IL8, IL10, and HMGB1, Blue Gene, Shanghai, China; for IL6 and TNF-α, R&D Systems, USA), according to the manufacturers’ instructions. The expression of IL1β, IL6, IL-8, IL10, TNF-α, and HMGB1 mRNAs was also measured at the same time with qPCR. Peripheral blood monocytes (PBMNs) were isolated with a method previously established in our laboratory [19]. Total RNA was extracted from the PBMNs with TRIzol Reagent (Invitrogen, USA) and reverse transcribed into cDNA with reverse transcriptase (TaKaRa, China) and a SYBR Green PCR Kit (TaKaRa, China), according to the manufacturer’s protocols. The transcription analysis of each sample was repeated at least three times. In this study, β-actin was used as the internal control. The primers used for the qPCRs are shown in Table 1.

**Table 1:** **Primer sequences for qRT-PCR**

Gene	Nucleotide Sequence (5′–3′)	Tm (°C)	Length (bp)
β-actin			
Forward	TGCGGGACATCAAGGAGAAG	57.4	216
Reverse	AGTTGAAGGTGGTCTCGTGG	57.4	
IL-1β			
Forward	TCTGCATGAGCTTTGTGCAAG	57.7	155
Reverse	ACAGGGCAGACTCGAATTCAAC	55.8	
IL-6			
Forward	TGTCGAGGCTGTGCAGATTAGT	57.7	142
Reverse	CATCCATCGTTCTGTGACTGC	57.6	
IL-8			
Forward	ACAGCAGTAACAACAACAAG	50.1	117
Reverse	GACCAGCACAGGAATGAG	50.2	
IL-10			
Forward	CGTGGAGGAGGTGAAGAGTG	55.4	178
Reverse	TTAGTAGAGTCGTCATCCTGGAAG	55.6	
TNF-α			
Forward	CGCTCTTCTGCCTACTGCACTTC	61.3	164
Reverse	CTGTCCCTCGGCTTTGACATT	57.6	
HMGB1			
Forward	GATCCTAAGAAGCCGAGAG	55.2	102
Reverse	GAAGTTGACTGAAGCATCTG	53.4	

### Determination of the effect of baicalin on the MAPK signalling pathway and apoptosis by western blotting

The effects of baicalin on the MAPK signalling pathway and on apoptosis were determined as previously described, with some minor modifications [18]. Briefly, the aortic vessels of the piglets were collected, and the total protein was extracted with a total protein extraction kit (Beyotime Biotechnology, China). The protein concentrations were measured with a BCA protein assay kit (Sigma, USA). The isolated total proteins were resolved with 12% SDS-PAGE and transferred onto polyvinylidene difluoride membranes, which were then blocked with 5% skimmed milk for 90 min and washed five times with Tris-buffered saline containing Tween 20 (TBST). The membranes were incubated with the appropriate antibodies or anti-β-actin antibody (Cell Signaling Technology, USA) for 14 h at 4 °C. After the membranes were washed five times with TBST, they were incubated with horseradish-peroxidase-linked goat anti-rabbit antibody (Proteintech, USA) for 30 min at 25 °C and visualized with ECL solution (Thermo Pierce ECL, USA). The protein expression levels of c-Jun N-terminal kinase (JNK), phosphorylated JNK (p-JNK), protein 38 (P38), p-P38, extracellular regulated protein kinases (ERK), p-ERK, and caspase 3 were determined with the FluorChem™ FC2 AIC system (Alpha Innotech, USA).

### Histopathology

Seven days after the *G. parasuis* SH0165 challenge, the brains and lungs of the surviving piglets from all groups were isolated, fixed in 10% neutral-buffered formalin, and embedded in paraffin. Tissue sections (4 μm thick) were stained with haematoxylin and eosin with the standard method and observed with light microscopy [25].

### Statistical analysis

The experimental data are shown as the means ± SDs. Differences between two groups were analysed with Student’s two-tailed *t* test. Survival analysis was performed with a log rank test. *p* values < 0.05 were considered significant; *p < 0.05 and **p < 0.01.

## Results

### Baicalin could protect piglets against *G. parasuis* challenge

**Figure 1:**
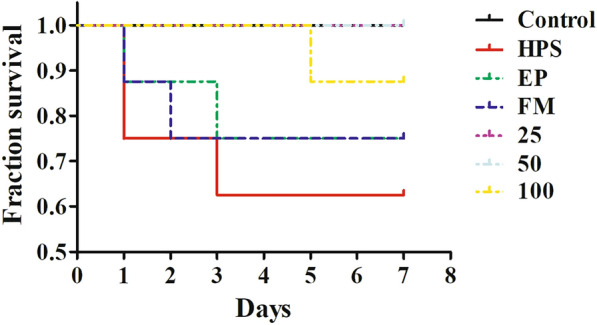
**Effects of baicalin on the protection of piglets against** ***G. parasuis*** **challenge. Before** ***G. parasuis*** **challenge, the piglets in the EP group were injected intraperitoneally with EP, and the piglets in the FM group were injected intramuscularly with FM**. After pretreatment with baicalin at concentrations of 25–100 mg/kg BW for 30 min, the piglets were challenged with 1 × 10^9^ CFU of *G. parasuis* strain SH0165. The mortality of the piglets was recorded. *G. parasuis*: HPS (the infection group); 25–100: the piglets were injected intramuscularly with baicalin at 25 mg/kg BW (treatment group 1), 50 mg/kg BW (treatment group 2), or 100 mg/kg BW (treatment group 3).

Baicalin provided 100%, 100%, and 87.5% protection to treatment group 1, treatment group 2, and treatment group 3, respectively, compared with that for the infection group, which was non-significant (*p *> 0.05, Figure 1). The mortality of the piglets in the infection group was 37.5% in the days after their challenge with *G. parasuis* strain SH0165, and treatment groups 1, 2, and 3 were protected during the observation period (Figure 1).

### Effects of baicalin on the release of cytokines in the serum of piglets challenged with *G. parasuis*

**Figure 2:**
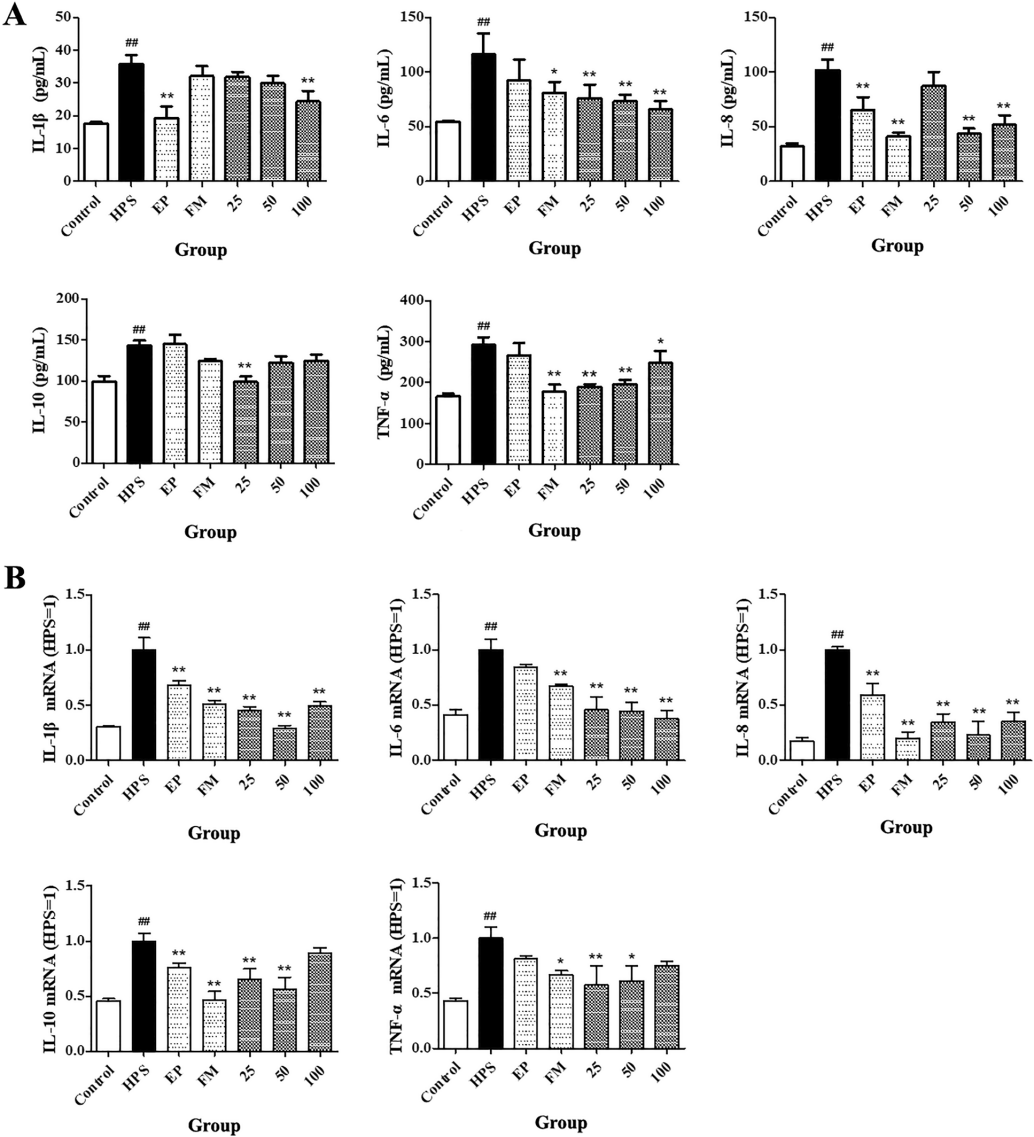
**Effects of baicalin on the release of cytokines into the serum of piglets challenged with** ***G. parasuis***. **A** Determination of cytokine IL1β, IL6, IL8, IL10, and TNF-α production by ELISA. **B** Determination of cytokine IL1β, IL6, IL8, IL10, and TNF-α expression levels by qPCR. Before *G. parasuis* challenge, the piglets in the EP group were injected intraperitoneally with EP, and the piglets in the FM group were injected intramuscularly with FM. At 48 h after *G. parasuis* challenge, blood samples were collected for analysis of the cytokines IL1β, IL6, IL8, IL10, and TNF-α. *G. parasuis*: HPS. 25–100: piglets were injected intramuscularly with baicalin at 25, 50, or 100 mg/kg BW. ^##^p < 0.01 vs control. *p < 0.05 vs the infection group, and **p < 0.01 vs the infection group.

The levels of IL1β, IL6, IL8, IL10, and TNF-α were significantly higher after *G. parasuis* challenge than in the control condition, as measured by ELISA (*p *< 0.01, Figure 2A). EP did not significantly reduce IL6, IL10, and TNF-α secretion (*p *> 0.05, Figure 2A), but EP significantly inhibited the production of IL1β and IL8 (*p *< 0.01, Figure 2A). FM did not significantly reduce IL1β and IL10 release (*p *> 0.05, Figure 2A), but FM significantly attenuated the production of IL6, IL8, and TNF-α, as measured by ELISA (*p *< 0.05 for IL6, and *p* < 0.01 for IL8 and TNF-α, Figure 2A). Baicalin at concentrations of 50–100 mg/kg BW reduced the secretion of IL6, IL8, and TNF-α, as measured by ELISA (*p *< 0.05 for TNF-α and *p* < 0.01 for IL6 and IL8, Figure 2A). *G. parasuis* also increased the expression of IL1β, IL6, IL8, IL10, and TNF-α mRNAs relative to that in the control condition (*p *< 0.01, Figure 2B). EP significantly attenuated the expression of IL1β, IL8, and IL10 (*p *< 0.01, Figure 2B). FM significantly reduced the expression of IL1β, IL6, IL8, IL10, and TNF-α (*p *< 0.05 for TNF-α, *p *< 0.01 for IL1β, IL6, IL8, and IL10, Figure 2B). Baicalin at concentrations of 25–50 mg/kg BW reduced the expression of IL1β, IL6, IL8, IL10, and TNF-α (*p *< 0.05 for TNF-α, *p *< 0.01 for IL1β, IL6, IL8, and IL10, Figure 2B).

### Effects of baicalin on the release of HMGB1 into the serum of piglets challenged with *G. parasuis*

**Figure 3:**
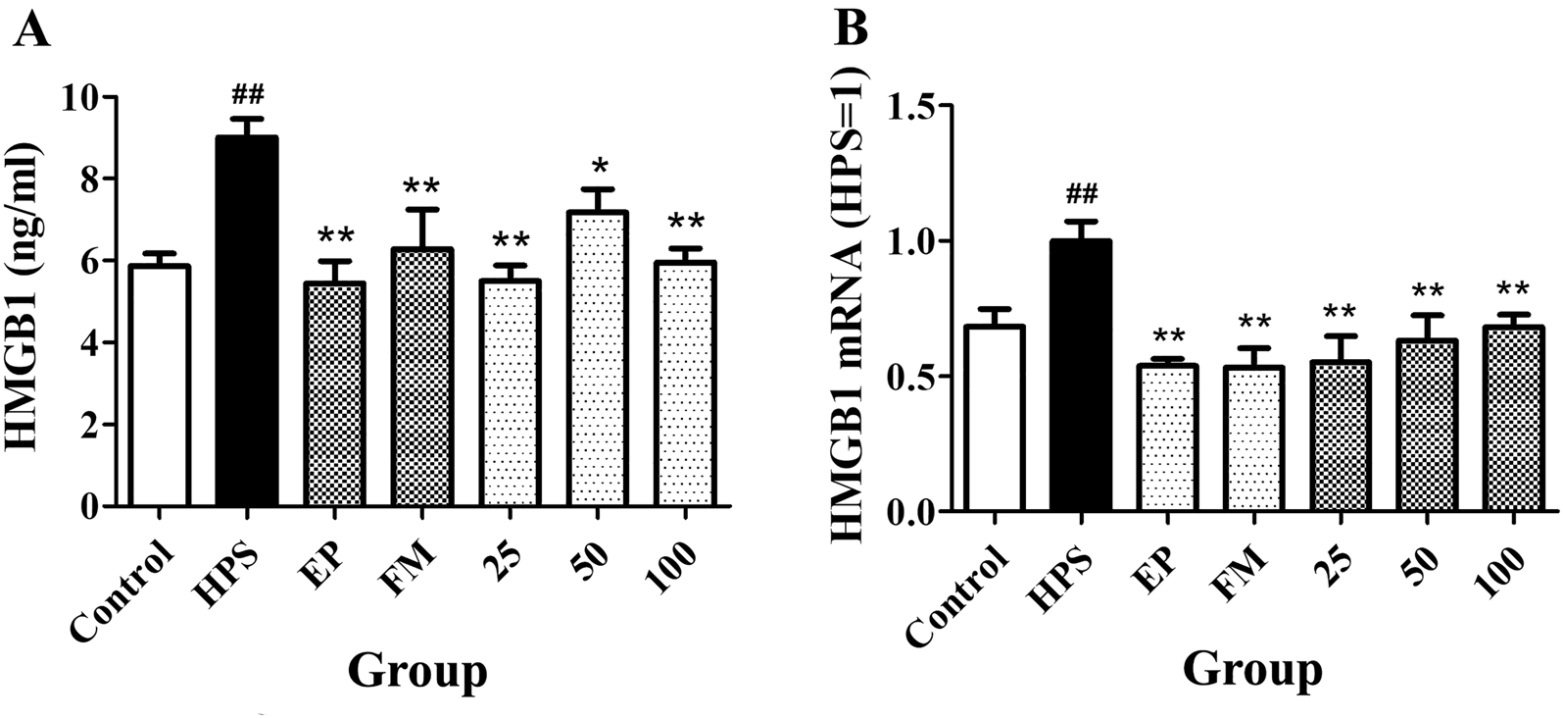
**Effects of baicalin on the release of HMGB1 into the serum of piglets challenged with** ***G. parasuis***. **A** Determination of HMGB1 production by ELISA. **B** Determination of the HMGB1 expression level by qPCR. Before *G. parasuis* challenge, the piglets in the EP group were injected intraperitoneally with EP, and the piglets in the FM group were injected intramuscularly with FM. At 48 h after *G. parasuis* challenge, blood samples were collected to measure the HMGB1 levels. *G. parasuis*: HPS. 25–100: the piglets were injected intramuscularly with baicalin at 25, 50, or 100 mg/kg BW. ^##^p < 0.01 vs control. *p < 0.05 vs the infection group, and **p < 0.01 vs the infection group.

After the piglets were challenged with *G. parasuis*, the levels of HMGB1 were significantly higher in the non-treated group than in the non-infected group, as measured by ELISA (*p *< 0.01, Figure 3A). EP and FM reduced the release of HMGB1 (*p *< 0.01, Figure 3A), and 25–100 mg/kg baicalin significantly reduced its release, according to ELISA results (*p *< 0.05 for 50, *p *< 0.01 for 25 and 100 mg/kg, Figure 3A). HMGB1 expression at the mRNA level was consistent with the ELISA results (*p *< 0.01, Figure 3B).

### Effects of baicalin on cell apoptosis induced by *G. parasuis*

**Figure 4:**
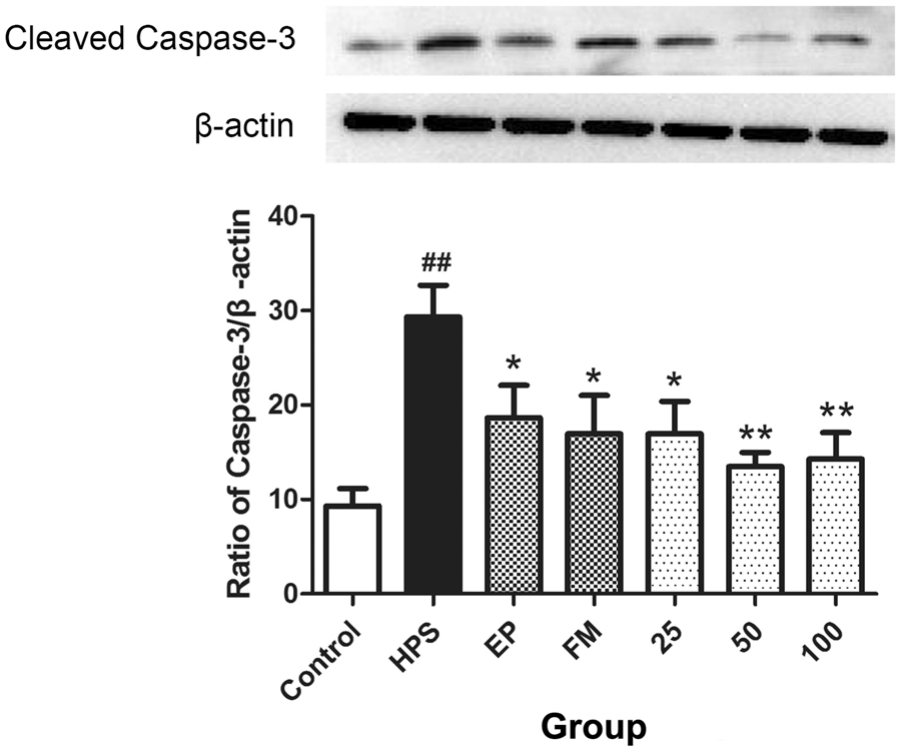
**Effects of baicalin on cell apoptosis induced by** ***G. parasuis***. Before *G. parasuis* challenge, the piglets in the EP group were injected intraperitoneally with EP, and the piglets in the FM group were injected intramuscularly with FM. Aortic vessels were collected, and the expression of caspase 3 was measured by western blotting. *G. parasuis*: HPS. 25–100: the piglets were injected intramuscularly with baicalin at 25, 50, or 100 mg/kg BW. ^##^p < 0.01 vs control. *p < 0.05 vs the infection group and **p < 0.01 vs the infection group.

To explore the effects of baicalin on the apoptosis induced by *G. parasuis*, the expression of caspase 3 was measured by western blotting. The results demonstrated that *G. parasuis* increased cell apoptosis after the piglets were challenged with the bacterium compared with the level in the control piglets (*p *< 0.01, Figure 4), but caspase 3 expression was inhibited in the EP and FM groups (*p *< 0.05, Figure 4). Baicalin (25–100 mg/kg BW) also attenuated cell apoptosis in piglets challenged with *G. parasuis* compared with that in the infection group piglets (*p* < 0.05 for 25, *p* < 0.01 for 50 and 100, Figure 4).

### Effects of baicalin on the MAPK signalling pathway activated by *G. parasuis*

**Figure 5:**
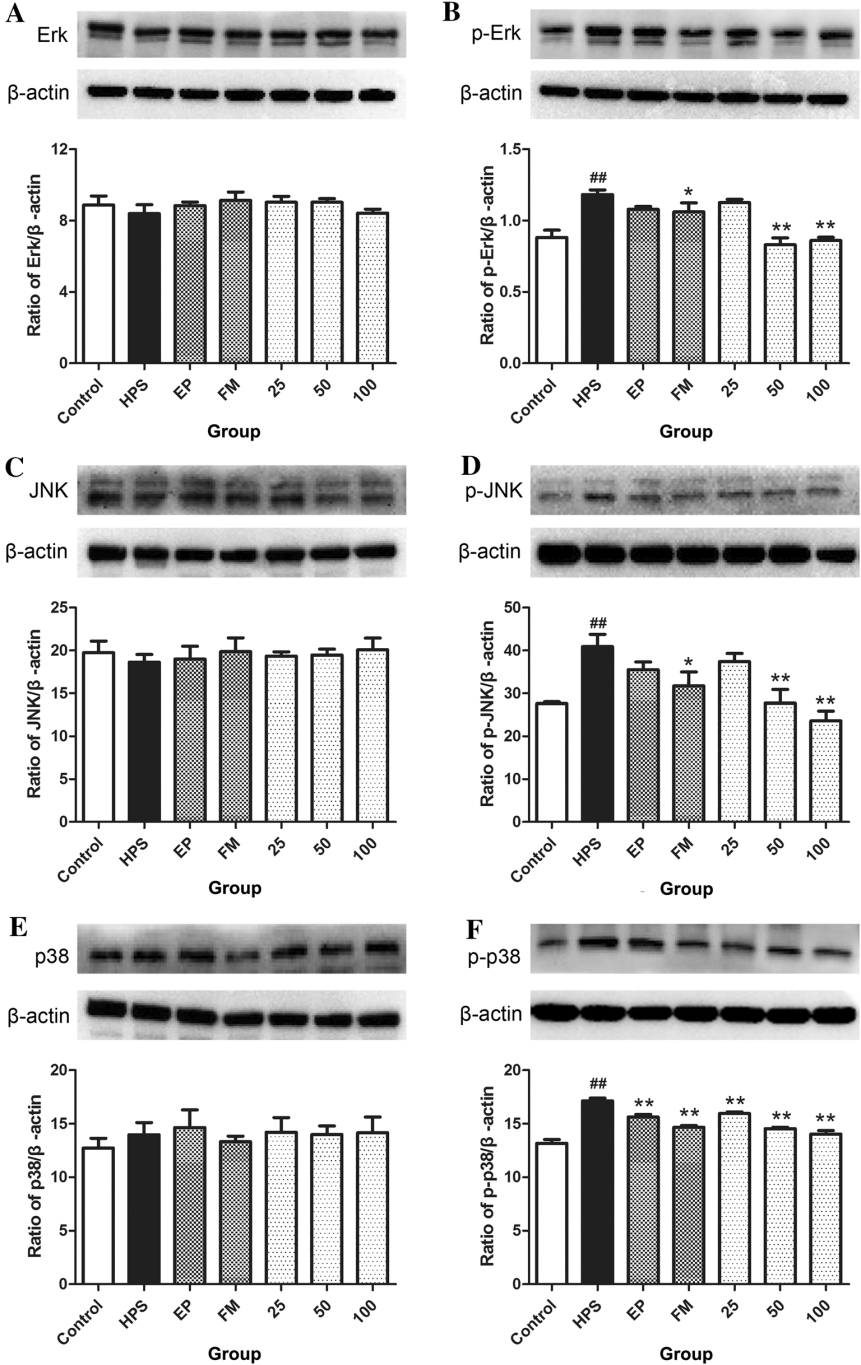
**Effects of baicalin on the MAPK signalling pathway activated by** ***G. parasuis***. Before *G. parasuis* challenge, the piglets in the EP group were injected intraperitoneally with EP, and the piglets in the FM group were injected intramuscularly with FM. Aortic vessels were collected, and the protein expression levels of ERK (**A**), p-ERK (**B**), JNK (**C**), p-JNK (**D**), P38 (**E**) and p-P38 (**F**) were determined. *G. parasuis*: HPS. 25–100: the piglets were injected intramuscularly with baicalin at 25, 50, or 100 mg/kg BW. ^##^p < 0.01 vs control. *p < 0.05 vs the infection group, and **p < 0.01 vs the infection group.

The data showed that *G. parasuis* promoted the phosphorylation of ERK, JNK, and P38 compared with that in the control condition(*p* < 0.05 for ERK and JNK, *p* < 0.01 for P38, Figure 5B, D, F), whereas the phosphorylation of ERK, JNK, and P38 in piglets challenged with *G. parasuis* was inhibited by FM (*p* < 0.05, Figure 5B, D, F), and EP attenuated the phosphorylation of P38 in these piglets (*p* < 0.01, Figure 5F). Baicalin (50–100 mg/kg BW) suppressed ERK, JNK and P38 phosphorylation in piglets infected with *G. parasuis* (*p* < 0.01, Figure 5B, D, F).

### Histopathological analysis

**Figure 6:**
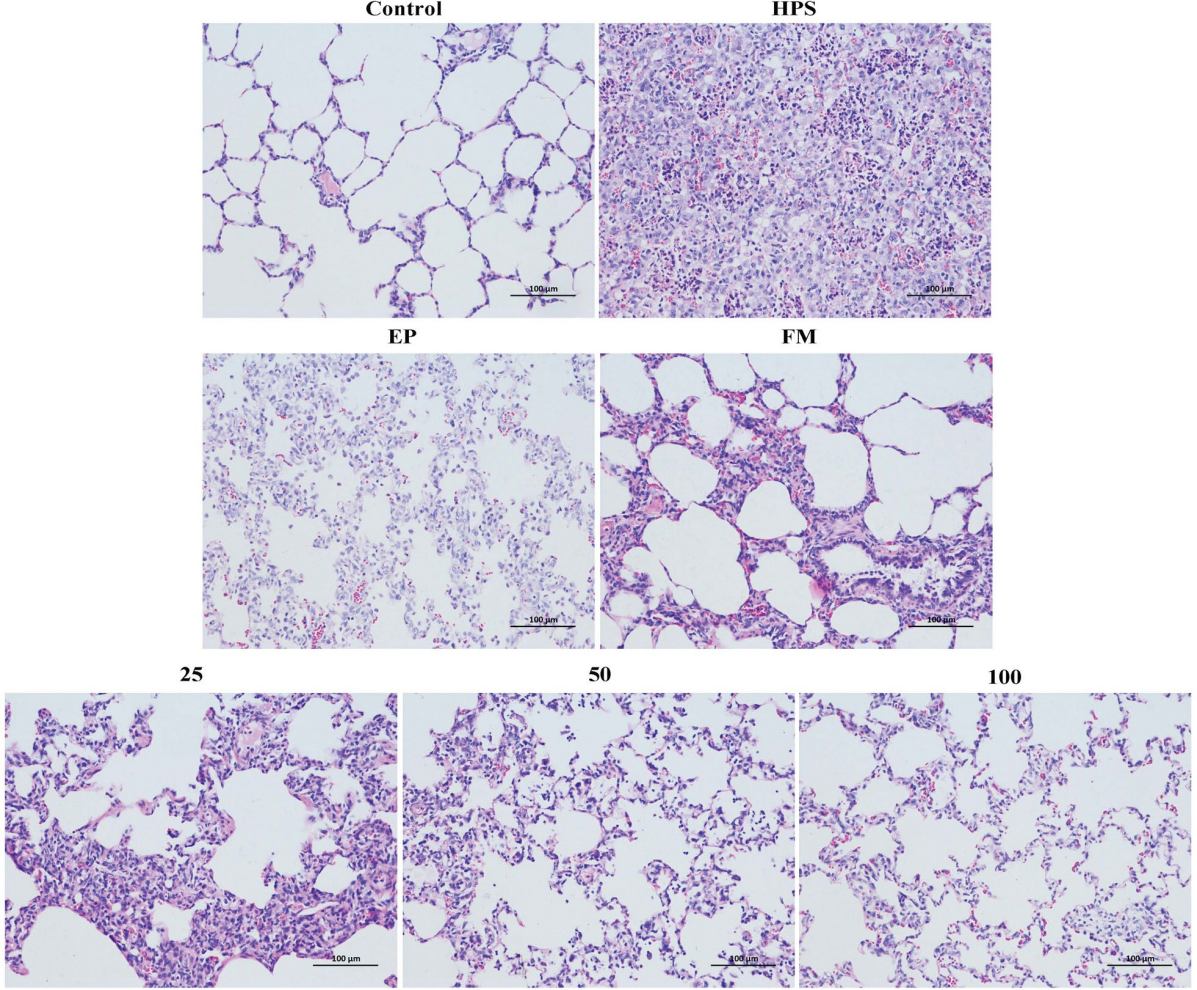
**Histopathological analysis of piglet lungs in the negative control group, the infection group, and the EP, FM, and baicalin treatment groups**. *G. parasuis*: HPS. 25–100: the piglets were injected intramuscularly with baicalin at 25, 50, or 100 mg/kg BW.

**Figure 7:**
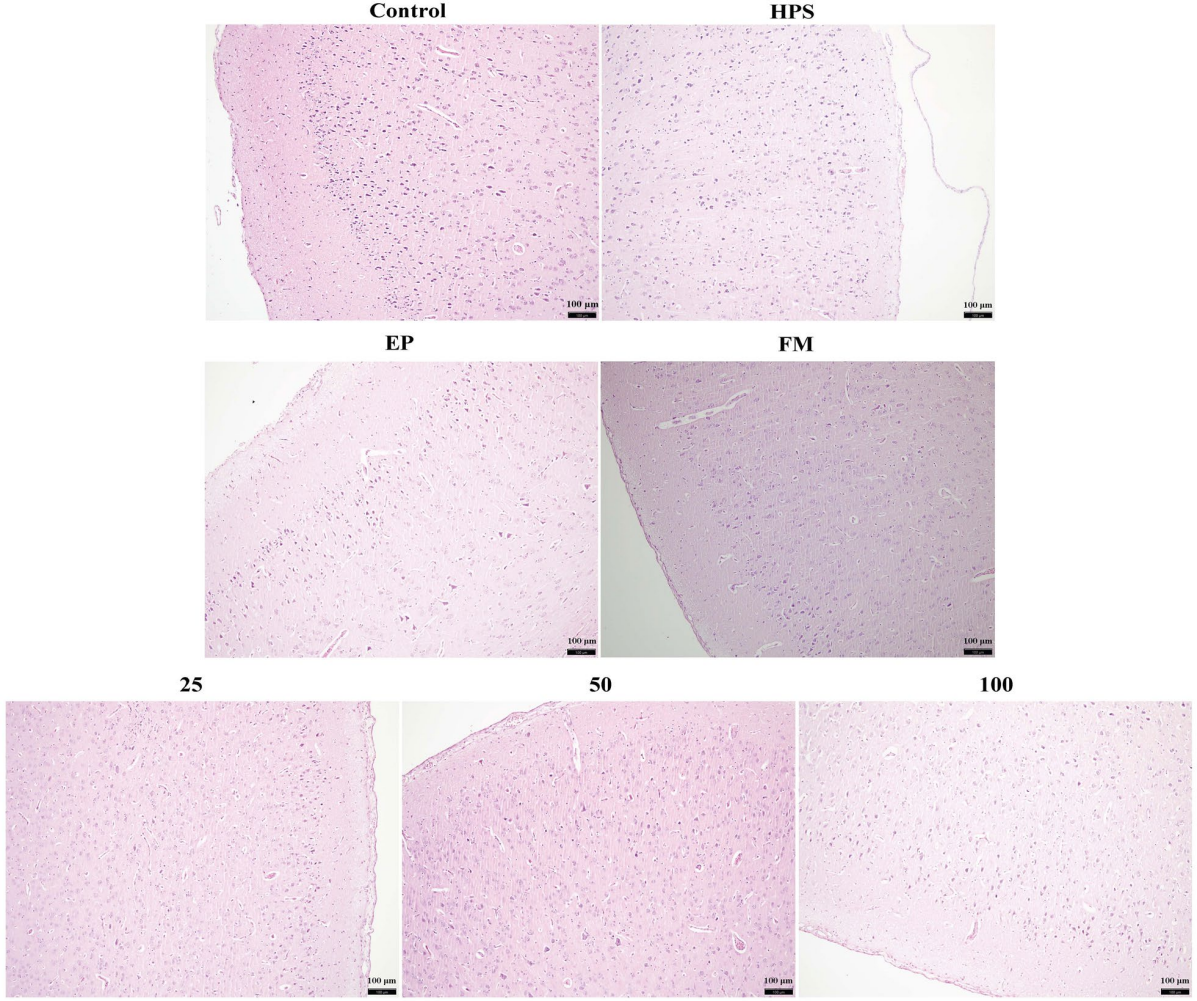
**Histopathological analysis of piglet brains in the negative control group, the infection group, and the EP, FM, and baicalin treatment groups**. *G. parasuis*: HPS. 25–100: the piglets were injected intramuscularly with baicalin at 25, 50, or 100 mg/kg BW.

The piglets from the *G. parasuis* infection group displayed severe pathological damage to their lung and brain tissues (Figures 6-HPS, 7-HPS). However, only mild tissue damage was detected in the surviving piglets of the EP, FM, and baicalin treatment groups (Figures 6 and 7). In the *G. parasuis* infection group, extensive proliferation of fibroblasts was detected in the lung parenchyma and massive neutrophil infiltration in the bronchioles (Figure 6-HPS). Inflammatory cell infiltration and aggregation were detected in the brains of the *G. parasuis* infection group (Figure 7-HPS). However, only minor histopathological damage was detected in the lungs or brains of the piglets in the EP, FM, and baicalin treatment groups (Figure 6-EP, FM, 25, 50, 100; Figure 7-EP, FM, 25, 50, 100).

## Discussion

In this study, we comprehensively investigated the anti-inflammatory efficacy of baicalin in piglets challenged with *G. parasuis* and the mechanism of its anti-inflammatory action. Our results demonstrate that baicalin could provide certain protection to piglets against *G. parasuis* challenge but not significantly, which might be related to the numbers of piglets. In the future, we will use more animals to study the protection of piglets against *G. parasuis* challenge afforded by baicalin. We also found that the anti-inflammatory effect of baicalin may involve the attenuation of cell apoptosis, the reduction in HMGB1 release, and the inhibition of the activation of the MAPK signalling pathway.

In this study, EP and FM were used as control drugs. Previous research has reported that EP reduced renal damage during renal diseases induced with methylglyoxal-derived advanced glycation end products [26]. EP reduced the release of IL8, TNF-α, IL6, and IL1β and attenuated apoptosis in LPS-induced inflammation in IPEC-J2 cells [27]. It effectively inhibited HMGB1 and receptor for advanced glycation end products (RAGE) expression, thus reducing the activity of the HMGB1–RAGE axis, and is therefore considered an inhibitor of HMGB1 [28]. Consistent with previous results, our data also showed that EP attenuates the expression of HMGB1 in piglets infected with *G. parasuis*. It has been documented that FM exerts significant anti-inflammatory effects in piglets and cattle [29, 30]. Flunixin meglumine also reduces the release of the cytokines IL1β, IL10, and TNF-α [31], so it is considered a good option for controlling inflammation-related diseases, such as *G. parasuis* infection.

Cytokines are important mediators of inflammation, immunity, and the pathological damage that occurs during the disease processes [32]. Previous research reported that IL1β, IL6, and IL8 were produced when pigs were challenged with *G. parasuis*, which could be considered one of the characteristics of *G. parasuis* infection [33, 34]. The expression levels of IL-10 and TNF-α in the spleen were upregulated after colostrum-deprived pigs were infected with *G. parasuis* [35]. Stimulation of Raw 264.7 macrophages with *G. parasuis* PotD could induce the production of IL1β, IL6, and TNF-α [36]. Therefore, these cytokines play important roles in the inflammatory damage caused by *G. parasuis* infection. Consequently, we quantified the expression of these cytokines (IL1β, IL6, IL8, IL10, and TNF-α) at both the mRNA and protein levels. Our results confirmed that *G. parasuis* induced IL1β, IL6, IL8, IL10, and TNF-α expression, and baicalin attenuated the production of all these cytokines in piglets challenged with *G. parasuis*, confirming that baicalin has anti-inflammatory activity. However, the special characteristics of these cytokines in the inflammatory damage that occurs during the pathogenesis of *G. parasuis* infection require further research.

HMGB1 is a ubiquitous nuclear protein that regulates the expression of key genes involved in the pathogenesis of many diseases [37]. Previous research has shown that HMGB1 released from mouse trophoblasts contributes to inflammation during *Brucella melitensis* infection [38]. HMGB1 is reported to induce signalling via the RAGE receptor and the HMGB1/RAGE/cathepsin B signalling pathway, which activates NLRP3-dependent coronary endothelial cell pyroptosis and thus plays an important role in the endothelial damage that occurs in Kawasaki disease [39]. The HMGB1—RAGE proinflammatory axis promotes vascular inflammation and endothelial cell apoptosis, resulting in vascular injury during acute limb ischaemia and reperfusion [40]. HMGB1 is also involved in acute lung injury triggered in mice by LPS by activating the RAGE/NF-κB, toll-like receptor 2 (TLR)2, and TLR4 signalling pathways [41]. HMGB1 also participates in systemic fibrotic diseases through the RAGE/MAPK and NF-κB signalling pathways [42]. In a previous study, we showed that *G. parasuis* stimulated the release of HMGB1 in piglet PBMNs [22] and induced the activation of the RAGE and MAPK signalling pathways in porcine aortic vascular endothelial cells [20]. Therefore, we investigated whether HMGB1 is released and the MAPK signalling pathway is activated in piglets after challenge with *G. parasuis*. Our results show that *G. parasuis* triggers the release of HMGB1 and the activation of the MAPK pathway in piglets and that baicalin significantly inhibits the production of HMGB1 and the activation of the MAPK pathway in these piglets. These data suggest that the HMGB1 and MAPK signalling pathways are involved in the inflammatory process or inflammatory damage during *G. parasuis* infection and could offer a novel therapeutic target to control Glässer’s disease. In future studies, different specific inhibitors of the MAPK signalling pathway will be used to further investigate the mechanism of baicalin action.

Natural target animals are more suitable for examining the functions, metabolism, and efficacy of substances directed against the specific pathogen in vivo [43]. Therefore, the target animal of *G. parasuis*, the piglet, was selected to study the function of baicalin. Our results demonstrate that baicalin could provide certain protection to piglets against *G. parasuis* challenge and may have utility as a new natural antibiotic-free drug to treat *G. parasuis* infection.

Cell apoptosis is a type of programmed cell death that may contribute to the orderly and efficient removal of damaged cells [44]. In a previous study, we also showed that baicalin inhibits the apoptosis induced by *G. parasuis* via the RAGE and MAPK signalling pathways in vascular endothelial cells [20]. However, whether baicalin attenuates apoptosis in piglets challenged with *G. parasuis* has not been investigated until now. Our results demonstrate that baicalin reduces cell apoptosis in piglets challenged with *G. parasuis*.

In conclusion, our results show that baicalin could protect piglets against *G. parasuis* challenge, which may be related to the anti-inflammatory effect it exerts by inhibiting the activation of HMGB1, cell apoptosis, and the MAPK signalling pathway. Our data extend our knowledge of the pharmacological properties of baicalin, which may have utility as a novel therapeutic drug for the prevention of Glässer’s disease.

## Data Availability

All data generated or analysed during this study are included in this published article.

## Abbreviations

*G. parasuis*: *Glaesserella parasuis*
IL: interleukin
TNF-α: tumour necrosis factor α
HMGB1: high mobility group box 1
MAPK: mitogen-activated protein kinase
PAM: porcine alveolar macrophage
LOS: lipooligosaccharide
NF-κB: nuclear factor κB
LPS: lipopolysaccharide
TUG1: taurine upregulated 1
APEC: avian pathogenic *Escherichia coli*
PAVECs: porcine aortic vascular endothelial cells
PMNPs: piglet mononuclear phagocytes
EP: ethyl pyruvate
FM: flunixin meglumine
BW: body weight
ELISA: enzyme-linked immunosorbent assay
qPCR: real-time quantitative PCR
PBMNs: peripheral blood monocytes
TBST: tris-buffered saline containing Tween 20
JNK: c-Jun N-terminal kinase
p-JNK: phosphorylated JNK
P38: protein 38
ERK: extracellular regulated protein kinase
RAGE: receptor for advanced glycation end products
TLR2: toll-like receptor 2
